# Supplementary material for: BCS-BEC crossover driven by small Fermi pockets of a high-Tc cuprate superconductor
Source: Nat Commun. 2026 Jun 2;17:4810. doi: 10.1038/s41467-026-73081-5 (PMC13230621; doi:10.1038/s41467-026-73081-5)
Supplement: Supplementary file 1 — Supplementary Information [file 41467_2026_73081_MOESM1_ESM.pdf]

Supplementary Information: BCS-BEC crossover driven by small Fermi pockets of a high-T_c cuprate superconductor

Junhyeok Jeong,[1] Yamato Enomoto,[2] Yoshimitsu Kohama,[1] Tomotaka Nakayama,[2] Kotaro Ando,[2] Kifu Kurokawa,[1] Soonsang Huh,[1] Zhuo Yang,[1] Toshihiro Nomura,[3] Matthew D. Watson,[4] Timur K. Kim,[4] Cephise Cacho,[4] Chun Lin,[5] Makoto Hashimoto,[5] Donghui Lu,[5] Shiro Sakai,[6] Takami Tohyama,[7] Kazuyasu Tokiwa,[2,*] and Takeshi Kondo[1,8,†]

[1]*Institute for Solid State Physics, The University of Tokyo, Kashiwa, Chiba 277-8581, Japan*

[2]*Department of Applied Electronics, Tokyo University of Science, Tokyo 125-8585, Japan*

[3]*Department of Physics, Faculty of Science, Shizuoka University, Shizuoka 422-8529, Japan*

[4]*Diamond Light Source Ltd, Harwell Science and Innovation Campus, Didcot, OX110DE, U.K.*

[5]*Stanford Synchrotron Radiation Lightsource, SLAC National Accelerator Laboratory, 2575 Sand Hill Road, Menlo Park, CA 94025, U.S.A.*

[6]*Faculty of Science and Technology, Sophia University, Tokyo 102-8554, Japan*

[7]*Department of Applied Physics, Tokyo University of Science, Tokyo 125-8585, Japan*

[8]*Trans-scale Quantum Science Institute, The University of Tokyo, Bunkyo-ku, Tokyo 113-0033, Japan*

(Dated: April 16, 2026)

NOTE 1: Laue image of four-layer cuprates.

Laue image of our single-crystal samples (Fig. S1), taken on the *ab*-plane in a back-reflection setup, shows a clear four-fold rotational symmetry. The absence of satellite reflections or spot splitting confirms that the crystal is free from structural modulation, which could otherwise complicate ARPES data in momentum space. The ring-shaped background intensities originate from the metal substrates used to glue the samples.

NOTE 2: Error of Fermi pocket area (or carrier density p) determined by quantum oscillation data.

Figures S2a-S2d present quantum-oscillation data of magnetic torque (de Haas-van Alphen effect) after background subtraction, which were used for fast Fourier transformation (FFT) analysis. The background signal was removed by fitting each raw torque signal with a cubic polynomial. The oscillation signals are plotted only for magnetic fields higher than the upper critical field (H_{c2}), where superconductivity is fully suppressed.

As indicated by the solid and dotted arrows in Fig. S2a, the quantum oscillation data exhibit the main and shoulder-like secondary peaks. This behavior can be modeled as the sum of two sinusoid components, indicating that the quantum oscillation data include two distinct frequencies. These two frequencies likely originate from bilayer splitting between adjacent inner CuO_2 planes in the crystal structure (see Fig. 1a). However, the secondary frequency is nearly degenerate with the main one; the two are so close that the FFT yields only a single peak (Fig. S2e).

Note that the small peak at low frequency (150 T) in Fig. S2e are not intrinsic, since this minor peak arises as an artifact from the background subtraction. Other weak peaks at higher frequency (*e.g.*, 760 T) correspond to higher harmonics of the main oscillation frequency.

The error in estimating the Fermi pocket area (or carrier density p) was assessed from the stability of the main peak near 380 T in the FFT spectra obtained from quantum-oscillation data at various temperatures (Figs. S2b-S2d). The full FFT spectra are shown in Figs. 1g-i of the main text. While the peak position shows slight temperature-dependent shifts, the variation is small within about 20 T, which roughly corresponds to the doping level p of 0.3%. We thus adopt $\pm 0.3\%$ of doping level as the experimental uncertainty. Note that such a small variation in the pocket area cannot be resolved in ARPES measurements due to the limited angular resolution (Fig. S2f).

* Corresponding author: Mail address: tokiwa@rs.tus.ac.jp
† Corresponding author: Mail address: kondo1215@issp.u-tokyo.ac.jp

NOTE 3: Estimation of the Fermi energy ε_F for UD71K, UD78K, and UD78K.

In the main text, the Fermi energy ($\varepsilon_F \sim 50$ meV) was estimated by fitting the band dispersion of UD71K. Here, we confirm that ε_F is comparable among the three samples: UD71K, UD74K, and UD78K, whose doping level differences are small within 0.6%.

Figures S3a-S3f show ARPES band dispersions near E_F for UD71K, UD74K, and UD78K. Fitting the occupied bands determines the top of the band, namely ε_F, to be approximately 50 meV in all cases. The data for UD71K (Figs. S3a,S3b) are identical to those in the main text. This is consistent with the small doping difference ($\pm 0.3\%$) discussed in **NOTE 2**. To estimate a possible variation of ε_F among the samples, we perform a tight-binding fit to the UD71K data and assume a rigid-band shift to simulate different doping levels.

In Figs. S3g,S3h, we show Fermi pockets for a tight-binding band, shifted in energy to correspond $p = 5.2\%$ (blue), 5.5% (black), and 5.8% (blue). The top of these bands is examined in Figs. S3i,S3j. ε_F varies only by ± 3 meV. This variation is small enough to conclude that the abrupt increase of the pairing strength from UD71K to UD78K is not due to an error in the estimation of ε_F, but an intrinsic feature in the four-layer cuprates.

NOTE 4: Tight-binding fitting to the Fermi pocket band.

In Figs. 1l and S3, we perform a tight-binding fit to the ARPES data to extract the Fermi energy ε_F. The same fitting parameters as those in the previous study on five-layer cuprates [1] are adopted here. Table 1 summarizes the fitting functions and the corresponding coefficients used to reproduce the band dispersion in the current four-layer cuprate observed by ARPES. The tight-binding band is expressed as:

$$\epsilon_{\mathbf{k}} = \sum_{i=0}^{6} t_i n_i \tag{1}$$

The resulting band dispersion calculated using the parameters in Table 1 is shown in Fig. S4.

TABLE 1. Tight-binding functions and coefficients used in fitting to our ARPES data.

i	n_i	t_i (eV)
0	1	-0.545
1	$\frac{1}{2}[\cos(k_x - k_y) + \cos(k_x + k_y)]$	0.1425
2	$\cos(k_x - k_y)\cos(k_x + k_y)$	-0.712
3	$\frac{1}{2}\{\cos[2(k_x - k_y)] + \cos[2(k_x + k_y)]\}$	-0.0215
4	$\frac{1}{2}\{\cos[2(k_x - k_y)]\cos(k_x + k_y) + \cos(k_x - k_y)\cos[2(k_x + k_y)]\}$	0.305
5	$\cos[2(k_x - k_y)]\cos[2(k_x + k_y)]$	-0.0955
6	$\frac{1}{2}\{\cos[3(k_x - k_y)] + \cos[3(k_x + k_y)]\}$	0.045

NOTE 5: Estimation of doping levels in the outer planes.

In the main text, the doping levels p of IP, which form the Fermi pocket, were determined precisely by ARPES and quantum oscillation measurements. The variation of p among the three samples (UD71K, UD74K, and UD78K) was found to be very small. Here, we determine the p values of the outer planes (OP), which form the Fermi arc in these samples, and find that the doping level lies in the underdoped regime with similarly small variation among the samples, consistent with the result for the Fermi pockets in IP.

Figures S5a-S5c displays the Fermi surface mappings measured by ARPES for UD71K, UD74K, and UD78K, respectively. Aside from a small Fermi pocket in IP, a Fermi arc for OP is clearly visible.

The doping level p of OP is estimated by fitting the Fermi arc determined from ARPES spectra (open circles in Figs. S5a-S5c) using a tight-binding model widely adopted for single- and double-layered cuprates [2]. We find that the estimated p values fall within the range of $\sim 9\%$ to $\sim 10\%$, indicating that all three samples are situated in the moderately underdoped regime. The small variation in p across the samples is consistent with that observed in the IP. Notably, $9 - 10\%$ is also the doping range where the superconducting (SC) gap magnitude reaches its maximum in single- and double-layered cuprates. This further emphasizes the abnormality of the large gap observed in the Fermi pockets at a rather small doping level of $p \sim 5.5\%$.

NOTE 6: Momentum dependence of the superconducting gap for all three samples.

In the main text, we demonstrated the SC gap in detail only for UD78K, which has the largest SC gap among our samples. Here, we present the detailed SC gaps for the other samples (UD71K and UD74K), together with that of UD78K.

Figures S6a-S6f show EDCs along the Fermi surface at the low temperature (10K), exhibiting the momentum dependence of the SC gaps in UD71K, UD74K, and UD78K. In each of the left panels (Figs. S6a,S6c,and S6e), the SC gaps of the inner plane (IP) and the outer plane (OP) are compared, whereas in each of the right panels, the SC gaps of inner plane (IP) and optimally doped $Bi_2Sr_2CaCu_2O_{8+\delta}$ (Bi2212) with the SC transition temperature (T_c) of 92 K [3] are compared. It is observed that the IP gaps are much larger than the OP gaps in all three samples. In addition, the IP gap always exceeds the Bi2212 gap: while the degree of exceeding is small in UD71K, it becomes apparent in UD74K and significant in UD78K.

These behaviors are demonstrated in Figs. S6g-S6i more clearly by plotting the SC gaps as a function of d-wave form $|\cos(k_x) - \cos(k_y)|/2$. Here, the gap magnitudes were determined from the peak position of symmetrized EDCs (not presented). The extrapolated SC gap to antinode Δ_0 is estimated by extrapolating the data around the nodal region as represented by the dotted line and arrow. Notably, the IP gap Δ_0^{IP} shows a dramatic increase from 37 meV to approximately 60 meV, in stark contrast with the OP gap Δ_0^{OP} showing rather moderate evolution. We emphasize that Δ_0~60 meV is one of the largest SC gaps reported in cuprate research by ARPES. Notably, the OP is paramagnetic, whereas the IP exhibits an AF order. Furthermore, the doping level of the OP ($p = 9\% \sim 10\%$) is closer to the optimal doping than that of IP ($p = 5.5\%$). Considering these seemingly unfavorable circumstances of IP, it is striking that the SC gap magnitude of IP is almost twice that of OP, reaching the largest gap in cuprates. This indicates that antiferromagnetic (AF) order does not compete with superconductivity, but rather coexists with it in an intimate relationship.

The extremely large gap magnitude is expected to yield a substantially large $\Delta_0/k_{\mathrm{B}}T_c$ ratio in the small pocket. Figure S6j plots the $\Delta_0/k_{\mathrm{B}}T_c$ ratio calculated for IPs and OPs in all three samples. Whereas the ratio for OP remains near 8.5, comparable to the previously reported value in underdoped Bi2212 [4], the ratio for IP rapidly increases from ~12 to ~18, which far exceeds that of the BCS prediction [5]. This implies that the IP with the small pocket achieves the strong coupling, rapidly approaching the BEC regime.

NOTE 7: Much sharper and better-defined Bogoliubov quasiparticle peaks in the small pocket band than in the Fermi arc band

In **NOTE6**, we compared the SC gaps between the pocket band in IP and the arc band in OP. Here, we focus on the spectral line shape and compare it between IP and OP (Fig. S7).

Figures S7a,S7b show the energy distribution curves (EDCs) and those symmetrized about the Fermi level for IP and OP, respectively, for the UD71K samples. The Fermi momentum (k_{F}) points measured are indicated in Fig. S7c with circles. For a fair comparison,

all spectra in Figs. S7a,S7b were normalized to their integrated area over the energy window of $[-50\ \text{meV}, 50\ \text{meV}]$. They are then plotted with the same value of a vertical offset. The spectra obtained from similar Fermi angles (ϕ) are shown side by side to facilitate direct comparison between IP (Fig. S7a) and OP (Fig. S7b). It is evident that the Bogoliubov quasiparticle peaks (or the SC coherent peaks) in the Fermi pocket of the IP are significantly sharper, and thus better defined, than those in the Fermi arc of the OP.

We further point out that, in the arc band of OP at $p \sim 10\%$ (Fig. S7b), the pseudogap with a higher energy scale than the SC gap deviates the gap symmetry from a simple d-wave form. The coherent peaks are almost absent at Fermi angles larger than 15°, where only broad spectra are observed (see shaded regions in Figs. S7b,S7c). As a result, the Fermi surface length contributing to the superfluid density in the arc band of OP is comparable to that in the pocket band of IP. Combined with the finding that the SC gaps are much larger in the pocket than in the arc, this implies that the contribution of the pocket to the superfluid density, and hence to superconductivity, is likely greater than that of the arc. These findings suggest that the small pocket plays a dominant role in achieving the high-T_c in the four-layer cuprates.

NOTE 8: Bulk T_c predominantly determined by the IP, which forms the Fermi pocket band.

We demonstrate that the SC transition temperature (T_c) in our samples is predominantly determined by the IP, which hosts the Fermi pocket band. This is particularly important for the Uemura plot (T_c/T_F) [6] of the four-layer cuprates. In the main text, we argue that T_c/T_F for IP reaches the theoretical upper limit of two-dimensional superconductivity ($T_c/T_F = 0.125$) [7]. This interpretation is based not only on the experimentally determined ε_F ($=k_B T_F$, where k_B is the Boltzmann constant and T_F is the Fermi temperature) for the pocket band in IP but also on the assumption that the T_c of the crystal is mainly decided by the superconductivity of IP, which forms the Fermi pocket. Here, we justify this assumption by comparing the evolution of the energy gap with temperature between IP and OP for UD78K, which has the largest SC gap among our samples.

Figures S8a,S8b show the temperature dependence of symmetrized EDCs measured at the same momentum cut (black line in Fig. S8c). Notably, the SC coherence peak in IP persists up to the bulk T_c=74 K (pink curves), while a finite pairing gap remains above T_c and closes at $T = 90$ K ($T_{\text{pair}}^{\text{IP}} = 90$ K; see the main text). On the other hand, the OP gap closes at the temperature lower than the bulk T_c (green curve in Fig. S8b). The spectral line shape may underestimate the gap-closing temperature; in such cases, an estimation of spectral weight filling at the Fermi level could be a more precise measure. Accordingly, we

performed such an analysis, but the result remained unchanged.

In Fig. S8d, we summarize the temperature dependence of the spectral energy gap for both CuO_2 planes (IP and OP). The data points are well fitted by a BCS-type gap function with the onset temperature $T_{\rm pair}$ estimated for each plane. $T_{\rm pair}^{\rm IP}$ is larger than the bulk $T_{\rm c}$, whereas $T_{\rm pair}^{\rm OP}$ is smaller than the bulk $T_{\rm c}$. We note that this corroborates the recent observations in other multilayer cuprates, suggesting that the clean IP determines the bulk $T_{\rm c}$ of the crystal [8, 9].

This set of results strongly supports our conclusion that the bulk $T_{\rm c}$ is predominantly determined by the IP. This conclusion is further supported by the following three observations discussed above:

(1) The superconducting gap magnitude (Δ_0) in the IPs is much larger than that of OPs, even reaching approximately twice in the UD78K sample (Fig. S6 and **NOTE 6**).

(2) The quasiparticle coherence peak is much sharper in IPs than that of OPs (Fig. S7 and **NOTE 7**). This implies that the pairs of IPs are more coherent with a longer lifetime.

(3) The coherent portion of the Fermi surface that contributes to superconductivity is substantially reduced in the OPs because a large fraction of it is dominated by ill-defined pseudogap spectra. Consequently, the number of carriers participating in the superfluid density becomes comparable to that of a small pocket in the IPs (**NOTE 8**).

Taken together, these findings suggest that, despite the lower overall carrier concentration in the IPs, their contribution to the total superfluid density is comparable to—or may even exceed—that of the OPs. This provides a justification for our classification of the four-layer cuprates on the Uemura plot in the main text.

NOTE 9: Comparison of pairing strength to other BCS-BEC crossover candidates.

A key parameter describing the BCS-BEC crossover is the ratio of the pair size ($\xi \approx 1/\delta k_{\rm F}$) to the interparticle spacing ($1/k_{\rm F}$). This ratio can be expressed as $1/k_{\rm F}\xi \approx \delta k_{\rm F}/k_{\rm F} \approx \Delta/\varepsilon_{\rm F}$. In the weak-coupling limit of the BCS superconductivity, a large number of pairs overlap with each other ($\xi \gg 1/k_{\rm F}$). In contrast, the pair size shrinks (or the interparticle spacing gets large) and pairs become non-overlapping ($\xi \ll 1/k_{\rm F}$) in the strong-coupling limit of BEC. Therefore, the ratio between these two characteristic lengths ($1/k_{\rm F}\xi$ or $\Delta/\varepsilon_{\rm F}$) serves as a dimensionless parameter determining the proximity to the BEC limit, and the BCS-BEC crossover regime typically has a ratio of approximately unity.

The widely acknowledged pair size ξ is the Ginzburg-Landau (GL) coherence length ($\xi_{\rm GL} = \sqrt{\Phi_0/2\pi\mu_0 H_{c2}[1-T/T_c]}$) and the Pippard coherence length ($\xi_{\rm Pippard} = \hbar v_{\rm F}/\pi\Delta$),

where Φ_0 is the flux quantum, H_{c2} is the out-of-plane upper critical field, and v_{F} is the Fermi velocity. The ratio $1/k_{\mathrm{F}}\xi_{\mathrm{Pippard}}$ is directly connected to the pairing strength $\Delta/\varepsilon_{\mathrm{F}}$ as follows, when a simple parabolic band structure is assumed:

$$\frac{1}{k_{\mathrm{F}}\xi_{\mathrm{Pippard}}} = \frac{\pi}{2}\frac{\Delta}{\varepsilon_{\mathrm{F}}} \tag{2}$$

In Table 2, we present data collected from various superconductors reported to lie in the BCS-BEC crossover regime [10–19] and compare those for four-layer cuprates determined by ARPES. The gap symmetry of cuprates is d-wave, in contrast to the s-wave symmetry in other superconductors on the list. Therefore, we used the SC gap and the Fermi wavenumber at the tip of the Fermi pocket as typical parameters for the four-layer cuprates. The coupling parameters ($\Delta/\varepsilon_{\mathrm{F}}$ and $1/k_{\mathrm{F}}\xi$) of the four-layer cuprates appear comparable to (or even larger than) those of other BCS-BEC crossover superconductors. These findings provide compelling evidence for the realization of a BCS-BEC crossover state in the clean IP of the four-layer cuprates.

NOTE 10: Determination of superconducting gap from the ARPES spectra.

The superconducting gaps shown in this study were obtained by fitting the symmetrized EDCs taken at the k_{F} points to the phenomenological model as follows [2]:

$$A(k_{\mathrm{F}}, \omega) = \frac{1}{\pi}\frac{\Sigma''}{(\omega - \Sigma')^2 + \Sigma''^2} \tag{3}$$

where the self-energy ($\Sigma = \Sigma' + i\Sigma''$) is expressed as:

$$\Sigma(k_{\mathrm{F}}, \omega) = -i\Gamma_1 + \frac{\Delta^2}{\omega + i\Gamma_0} \tag{4}$$

Here, Γ_1 is the single-particle scattering rate and Γ_0 is the inverse-pair lifetime.

In Fig. S9, we present a representative gap analysis of our ARPES data. Figs. S9b and S9c are the raw and symmetrized (Sym.) EDCs of the UD78K sample, taken at the k_{F} point that is slightly away from the pocket tip (Fig. S9a). After symmetrization, we fit the spectra with the phenomenological model, which matches the data well (Fig. S9c).

NOTE 11: Temperature-dependence of Bogoliubov flat band.

In Fig. 4, we show the emergence of the Bogoliubov flat band in UD78K, a signature of BCS-BEC crossover. Here, we show the temperature-dependence of the Bogoliubov flat band. Figures S10a-d present ARPES images of UD78K sample, taken along the antiferromagnetic zone boundary (AFZB) at several temperatures spanning below and well above ($T_{\mathrm{pair}} \sim 100$ K). The Bogoliubov flat band is clearly resolved at low temperature ($T = 7$ K).

With increasing temperature, the feature progressively broadens and becomes less distinct, and it is no longer discernible at $T = 175$ K.

To further clarify this evolution, we plot temperature-dependent EDCs measured at the pocket center ($k = 0$) in Fig. S10e. The low-temperature spectrum exhibits a pronounced peak-dip-hump structure, a characteristic signature of strong mode coupling commonly observed in cuprates. Although this feature persists into the normal state, it weakens at elevated temperatures and disappears around $T = 175$ K.

Notably, a similar flat band is not observed in the OPs, which are disordered due to their direct proximity to the dopant layer. This contrast supports that the Bogoliubov flat band observed in the UD78K sample is an intrinsic electronic property.

NOTE 12: Superconducting gap along AFZB.

In Figs. 2e-f, we demonstrated the superconducting gap at the pocket tip, where it reaches its maximum value in the IPs of each sample. To further corroborate this gap magnitude, we examine the data along the AFZB cut, where k_F is unambiguously identified from the gap minimum.

Figures S11a and S11b show ARPES intensity map and the corresponding EDCs measured for UD78K below T_c ($T = 7$ K): the same data as in Figs. 4f, j. To clarify the energy gap, we zoom into the spectra near E_F in Fig. S11. The minimum gap, observed at k_F, is estimated to be approximately 30 meV (solid blue line in Fig. S11c) from the peak positions. These data confirm that a large superconducting gap of ~ 30 meV opens at the pocket tip.

NOTE 13: *d*-wave superconducting gap symmetry on the outer side of the small Fermi pocket.

Previous ARPES studies on the five-layer system have established that the superconducting gap exhibits *d*-wave symmetry on both the inner and outer sides of the small Fermi pocket [1]. Here, we confirm that the same behavior holds for the present four-layer material.

Figures S12b and S12c show representative EDCs of UD74K measured at the k_F points on the inner and outer sides of the pocket, respectively (marked by magenta and blue circles in Fig. S12a). Although the spectral intensity is much weaker on the outer side, reliable peak positions can still be extracted.

The extracted gap values follow a clear *d*-wave momentum dependence: the gap reaches its maximum near the pocket tip and decreases continuously toward the nodal direction, vanishing at the nodal cut. As shown in Fig. S12d, the gap magnitudes on the outer side quantitatively agree with those on the inner side within experimental uncertainty. These results confirm that the superconducting gap preserves *d*-wave symmetry over the entire

pocket, including its outer region.

NOTE 14: Effective masses of the small Fermi pocket: comparison between ARPES and quantum oscillation measurements

In the main text, we showed that the Fermi pocket size obtained by ARPES is fully consistent with that determined from quantum oscillation measurements. Here, we further compare the effective masses obtained from the two techniques.

In principle, ARPES is well-suited to evaluate the effective mass from the slope of the band dispersion. However, the mass variation observed in quantum oscillations is very small (see Figs. 1g-i), changing from $m^* \sim 0.82\,m_0$ in UD71K to $\sim 0.91\,m_0$ in UD78K (Figs. 1g-i). This corresponds to only a subtle modification of the band slope.

In cuprates, the intrinsic spectral linewidth is substantially broader than the change in dispersion slope associated with such a mass variation. As a result, resolving this small difference in effective mass directly from ARPES data is extremely challenging. To illustrate this limitation, we model a simple ellipsoidal band dispersion using the effective masses determined from quantum oscillation measurements and show that small mass variations are indistinguishable within the finite spectral linewidth. Nevertheless, we find that the overall dispersion agrees remarkably well with ARPES, demonstrating that ARPES and quantum oscillation measurements are quantitatively consistent.

We consider a two-dimensional elliptical parabolic band $\varepsilon(\boldsymbol{k})$ as a function of momenta k^{Node} and k^{AFZB} setting axes along the nodal and AFZB directions:

$$\varepsilon(\boldsymbol{k}) = \frac{\hbar^2}{2}\left(\frac{(k_{\mathrm{Node}})^2}{m_{\mathrm{Node}}} + \frac{(k_{\mathrm{AFZB}})^2}{m_{\mathrm{AFZB}}}\right), \quad \text{with} \quad \boldsymbol{k} = (k^{\mathrm{Node}}, k^{\mathrm{AFZB}}), \tag{5}$$

where m_{Node} and m_{AFZB} are the effective masses along the nodal and AFZB axes. The constant-energy contour at $E = \varepsilon_{\mathrm{F}}$ is obtained from

$$\frac{k^{\mathrm{Node}}}{k_{\mathrm{F}}^{\mathrm{Node}}} + \frac{k^{\mathrm{AFZB}}}{k_{\mathrm{F}}^{\mathrm{AFZB}}} = 1, \tag{6}$$

with

$$k_{\mathrm{F}}^{\mathrm{Node}} = \sqrt{\frac{2m_{\mathrm{Node}}\varepsilon_{\mathrm{F}}}{\hbar^2}}, \quad k_{\mathrm{F}}^{\mathrm{AFZB}} = \sqrt{\frac{2m_{\mathrm{AFZB}}\varepsilon_{\mathrm{F}}}{\hbar^2}}. \tag{7}$$

Here, $k_{\mathrm{F}}^{\mathrm{Node}}$ and $k_{\mathrm{F}}^{\mathrm{AFZB}}$ correspond to the half size of the ellipsoidal Fermi pocket in the nodal and AFZB direction, respectively, as illustrated in Fig. S13a.

The average mass along the Fermi surface m^*, or the cyclotron mass in quantum oscillation, is expressed as

$$m^* = \sqrt{m_{\mathrm{Node}} m_{\mathrm{AFZB}}} \tag{8}$$

Using $k_{\mathrm{F}}^{\mathrm{Node}}$ and $k_{\mathrm{F}}^{\mathrm{AFZB}}$ obtained from the tight-binding fit to our ARPES data (Supplementary NOTE 4) and $\varepsilon_{\mathrm{F}} = 50$ meV, we calculated the elliptical parabolic bands with three different masses: $m^* = 0.82\ m_0, 0.86\ m_0$, and $0.91\ m_0$ for UD71K, UD74K, and UD78K, respectively. For comparison, we overlay these curves on the ARPES dispersion for UD74K along the nodal and AFZB momentum cuts in Figs. S13b and S13c. Notably, the difference in the calculated band slopes for the three effective masses is very small (see the inset of Figs. S13b and S13c). The expected variation is far smaller than the intrinsic spectral linewidth, so that it cannot be resolved by ARPES. Nevertheless, the calculated dispersions reproduce the ARPES data remarkably well, demonstrating that the ARPES and quantum oscillation results are quantitatively consistent.

NOTE 15: Determination of errors in the pairing temperature.

For UD71K and UD74K samples, the determination of T_{pair} is straightforward because the quasiparticle peaks clearly persist above T_{c}. In these samples, we can determine the gap closing temperature (or T_{pair}) as the temperature where two peaks merge to one peak (Figs. 3a, b). Since the peak evolution is well resolved over multiple temperature points, the uncertainty in T_{pair} is small. Supplementary Fig. S8 presents the analysis for UD74K, where the gap-closing behavior is unambiguous and yields a relatively precise estimate of T_{pair} .

In contrast, UD78K does not show a clear peak-merging behavior at high temperatures. The spectra remain broadened due to a pseudogap that competes with superconductivity. In this case, distinguishing the pairing temperature T_{pair} from the pseudogap temperature T^* requires a careful analysis of the temperature-dependent spectral loss at E_{F}. Specifically, we identify T_{pair} as the temperature below which the spectral-weight loss upon cooling deviates from the pseudogap-derived linear behavior, following the procedure established in a previous ARPES study [20].

Based on this analysis, we estimate the uncertainty in T_{pair} for UD78K to be ±3 K. To clarify this point, we present the spectral-weight analysis for UD78K in Fig. S14. Figure S14a reproduces the data of Fig. 3e. In Fig. S14b, we subtract the linear fit to experimental data in the high-temperature region from the overall data points. This procedure enhances the visibility of the deviation from the high-temperature linear behavior upon cooling, which we associate with the onset of pair formation [20]. The deviation occurs at approximately 100 K. Considering (i) the residuals of the linear fit and (ii) the finite temperature interval over which the deviation develops, we estimate an uncertainty of ±3 K, as indicated by the

shaded region in Fig. S14b.

TABLE 2. Experimental data collected from various BCS-BEC crossover candidates. FeSe(ML)/STO, monolayer FeSe grown on the $SrTiO_3$ substrate; MATBG, magic-angle twisted bilayer graphene.

Materials	T_c (K)	Δ	ε_F (meV)	Δ/ε_F	k_F (nm^{-1})	$1/k_F\,\xi_{Pippard}$	$\xi_{Pippard}$ (nm)	$1/k_F\,\xi_{GL}$	ξ_{GL} (nm)
FeSe(ML)/STO [10, 11]	38	15	60	0.25	2.3	1.2	0.36	···	···
$FeSe_xTe_{1-x}$ [12–14]	12	3	6	0.5	1.2	···	···	2.21	0.38
$FeSe_{1-x}S_x$ [15]	4	1.6	14	0.11	···	···	···	···	···
Li_xZrNCl [16]	15.9	4.08	11.3	0.36	0.52	3.42	0.56	5.41	0.36
κ-$(BEDT\text{-}TTF)_4HgBr$ [17]	4.2	···	···	···	1	···	···	3	0.33
4-layer cuprates (F0234)	71	15	50	0.3	1.41	0.47	1.51	···	···
	74	19	50	0.38	1.37	0.60	1.22	···	···
	78	30	50	0.6	1.49	0.94	0.71	···	···
MATBG [18, 19]	1	1.4	1.7	0.8	···	···	···	···	···

References

[1] Kunisada, S. *et al.* Observation of small Fermi pockets protected by clean CuO_2 sheets of a high-T_c superconductor. *Science* **838**, 833–838 (2020).

[2] Norman, M., Randeria, M., Ding, H. & Campuzano, J. Phenomenological models for the gap anisotropy of $Bi_2Sr_2CaCu2O8$ as measured by angle-resolved photoemission spectroscopy. *Phys. Rev. B* **52**, 615 (1995).

[3] Kondo, T. *et al.* Point nodes persisting far beyond T_c in Bi2212. *Nat. Commun.* **6**, 7699 (2015).

[4] Anzai, H. *et al.* Relation between the nodal and antinodal gap and critical temperature in superconducting Bi2212. *Nat. Commun.* **4**, 1–7 (2013).

[5] Won, H. & Maki, K. *d*-wave superconductor as a model of high-T_c superconductors. *Phys. Rev. B* **49**, 1397 (1994).

[6] Uemura, Y. J. Condensation, excitation, pairing, and superfluid density in high-T_c superconductors: the magnetic resonance mode as a roton analogue and a possiblespin-mediated pairing. *Journal of Physics: Condensed Matter* **16**, S4515 (2004).

[7] Hazra, T., Verma, N. & Randeria, M. Bounds on the Superconducting Transition Temperature: Applications to Twisted Bilayer Graphene and Cold Atoms. *Phys. Rev. X* **9**, 031049 (2019).

[8] Sun, X. *et al.* High Temperature Superconductivity Dominated by Inner Underdoped CuO_2 Planes in Quadruple-Layer Cuprate (Cu, C)$Ba_2Ca_3Cu_4O_{11+\delta}$. *arXiv preprint arXiv:2507.03921* (2025).

[9] Jeong, J. *et al.* Superconducting coherence boosted by outer-layer metallic screening in multilayered cuprates. *arXiv preprint arXiv:2507.23260* (2025).

[10] Liu, D. *et al.* Electronic origin of high-temperature superconductivity in single-layer FeSe superconductor. *Nat. Commun.* **3**, 931 (2012).

[11] Zhang, S. *et al.* Enhanced superconducting state in FeSe/$SrTiO_3$ by a dynamic interfacial polaron mechanism. *Phys. Rev. Lett.* **122**, 066802 (2019).

[12] Lubashevsky, Y., Lahoud, E., Chashka, K., Podolsky, D. & Kanigel, A. Shallow pockets and very strong coupling superconductivity in $FeSe_xTe_{1-x}$. *Nat. Phys.* **8**, 309–312 (2012).

[13] Rinott, S. *et al.* Tuning across the BCS-BEC crossover in the multiband superconductor $Fe_{1+y}Se_xTe_{1-x}$: An angle-resolved photoemission study. *Science Advances* **3**, e1602372 (2017).

[14] Shruti, S., Sharma, G. & Patnaik, S. Anisotropy in upper critical field of $FeTe_{0.55}Se_{0.45}$. In *AIP Conference Proceedings*, vol. 1665 (AIP Publishing, 2015). URL `https://pubs.aip.org/aip/acp/article-abstract/1665/1/130030/883699`.

[15] Hashimoto, T. *et al.* Bose-Einstein condensation superconductivity induced by disappearance

of the nematic state. *Science Advances* **6**, eabb9052 (2020).

[16] Nakagawa, Y. *et al.* Gate-controlled BCS-BEC crossover in a two-dimensional superconductor. *Science* **372**, 190–195 (2021).

[17] Suzuki, Y. *et al.* Mott-driven BEC-BCS crossover in a doped spin liquid candidate κ-(BEDT-TTF)$_4$Hg$_{2.89}$Br$_8$. *Phys. Rev. X* **12**, 011016 (2022).

[18] Cao, Y. *et al.* Unconventional superconductivity in magic-angle graphene superlattices. *Nature* **556**, 43–50 (2018).

[19] Oh, M. *et al.* Evidence for unconventional superconductivity in twisted bilayer graphene. *Nature* **600**, 240–245 (2021).

[20] Kondo, T. *et al.* Disentangling Cooper-pair formation above the transition temperature from the pseudogap state in the cuprates. *Nat. Phys.* **7**, 21–25 (2010).

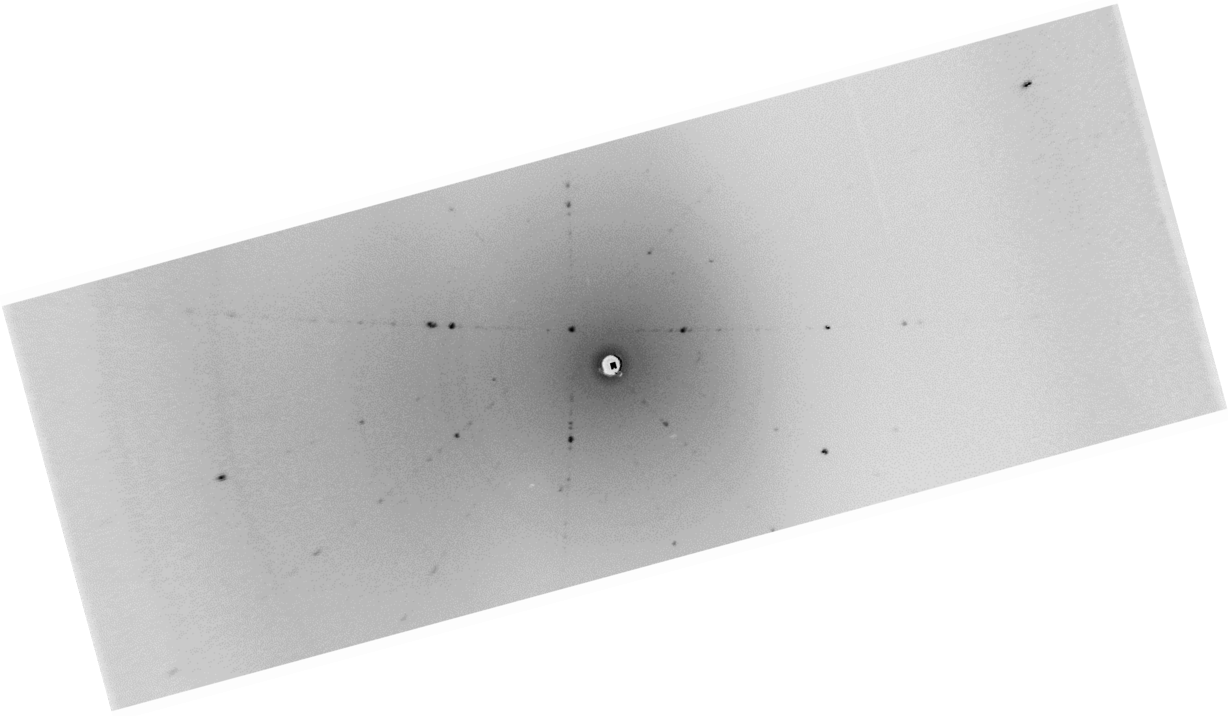

FIG. S1. **Laue image of a single crystal $Ba_2Ca_3Cu_4O_8(F,O)_2$.** Back-reflection Laue image of a four-layer cuprate sample, showing clear four-fold rotational symmetry without any sign of structural modulation.

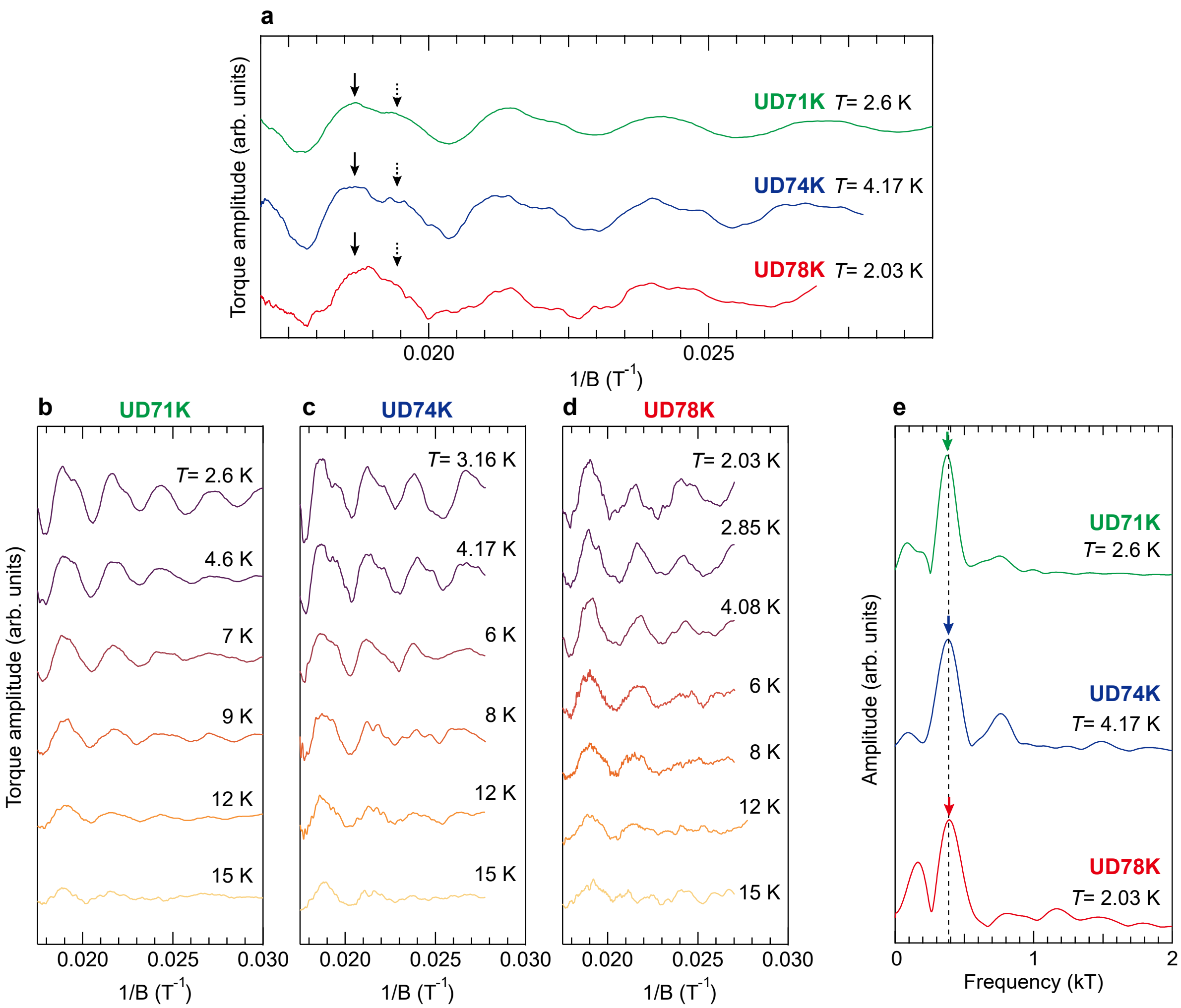

FIG. S2. **Raw magnetic torque signals and their Fourier analysis. a,** Selected magnetic torque signals of UD71K, UD74K, and UD78K from (**b-d**). The presence of two oscillation components is indicated by black solid and dashed arrows. **b-d,** Magnetic torque signals of UD71K, UD74K, and UD78K measured at various temperatures. A cubic background was subtracted from the raw data. The results after FFT analysis are shown in the main text Fig. 2**g-i**. **e,** FFT spectra of the curves in (**a**), showing a single frequency peak near 380 T for all samples, confirming that the two components are nearly degenerate in frequency.

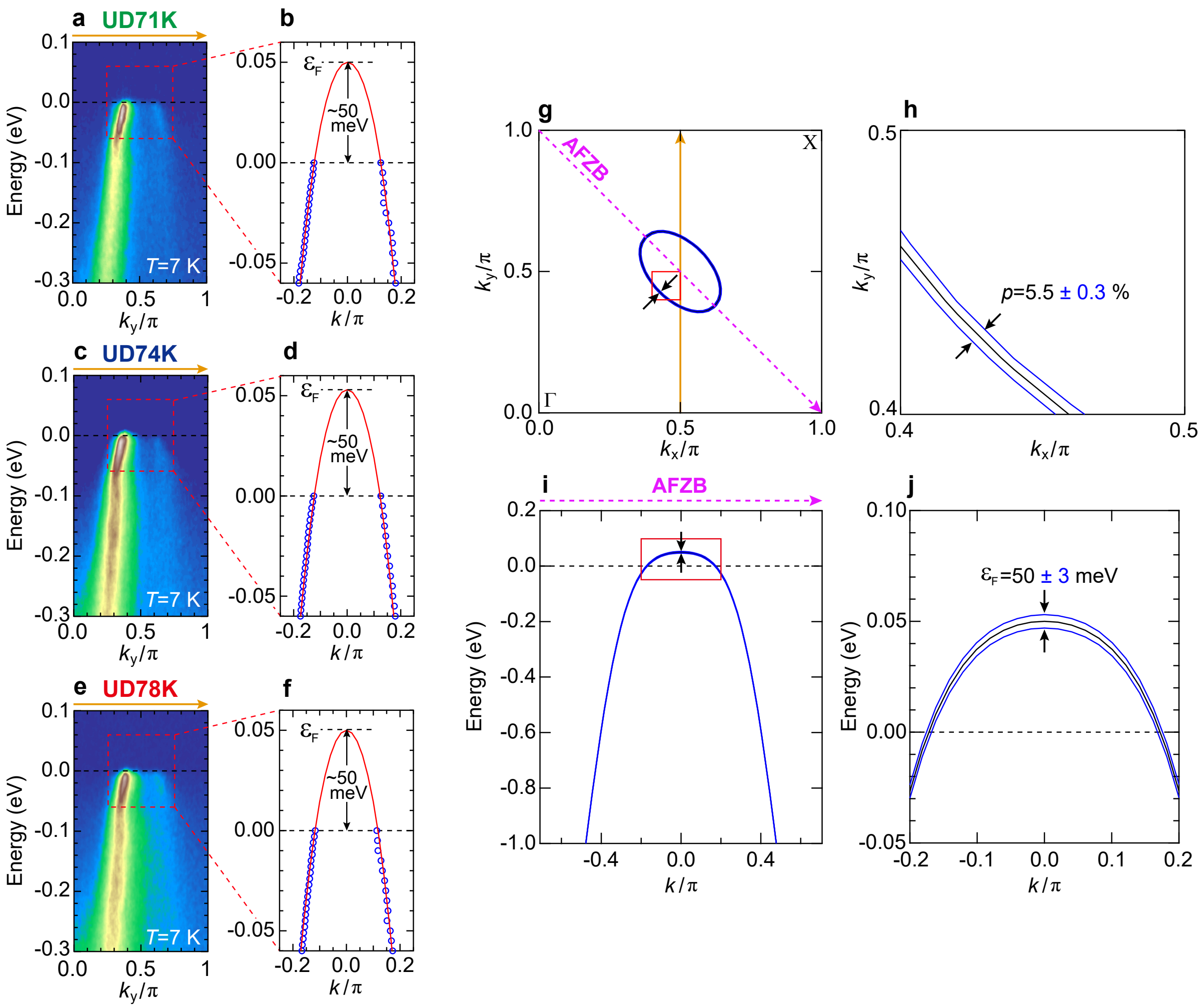

FIG. S3. **Estimation of the Fermi energy ε_F and its error. a,** ARPES band dispersion of the IP in UD71K, measured along the orange arrow in (**g**). **b,** Band dispersion determined from the peak positions of the momentum distribution curves (MDCs) in (**a**). The Fermi energy ε_F (black arrow) is obtained by fitting the dispersion (red solid line) using a tight-binding model. (**c-f,**) Same measurement as (**a,b**), but for UD74K and UD78K, respectively. (**g,**) Small Fermi pockets with three different carrier densities: $p = 5.2\%$ (blue), 5.5% (black), and 5.8% (blue). The tight-binding fit to the ARPES data with $p = 5.5\%$ was shifted in energy to simulate the Fermi pocket of $p = 5.2\%$ and 5.8%. (**h,**) Enlarged view of the red box region in (**g**). (**i,**) Band dispersion for (**a**), plotted along the antiferromagnetic zone boundary (AFZB, pink dashed arrow in (**g**)). (**j,**) Enlarged view of the boxed region in (**i**), showing that a doping variation of $\pm 0.3\%$ corresponds to a shift in ε_F of approximately ± 3 meV ($\varepsilon_F = 50 \pm 3$ meV).

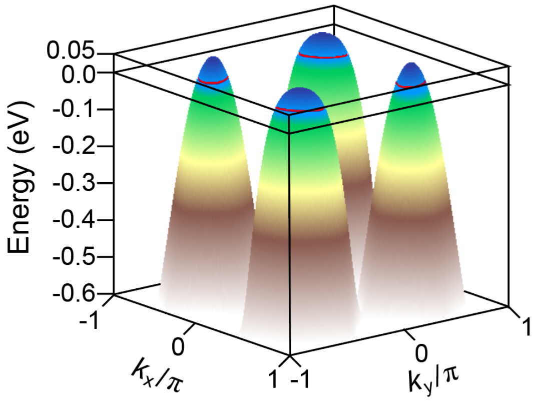

FIG. S4. **Tight-binding band of the Fermi pocket.** Band dispersion determined by tight-binding fitting to our ARPES data.

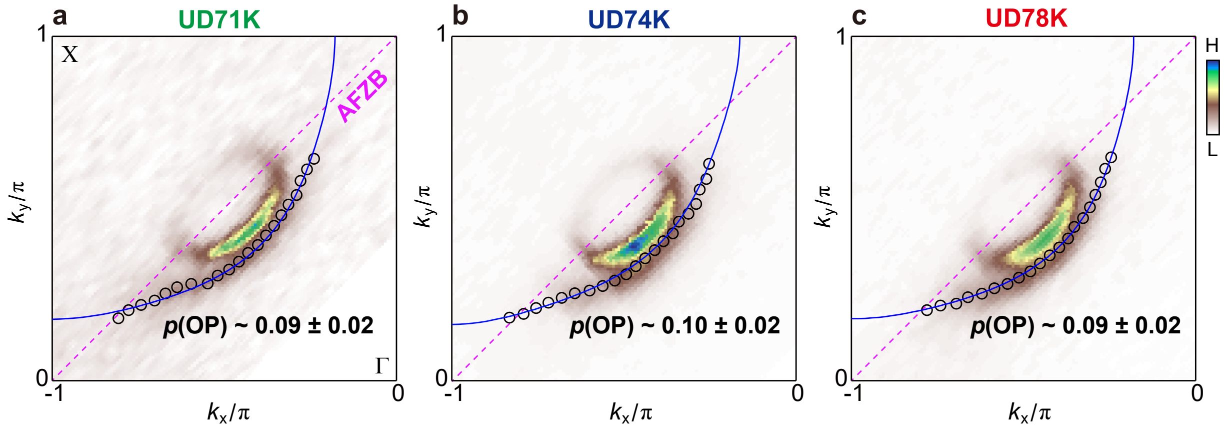

FIG. S5. **Doping level estimation for the outer planes (OP) in three samples. a,** Fermi surface mapping of UD71K measured by ARPES. The k_F points of the OP band (Fermi arc) are indicated by black circles. The blue line represents the tight-binding fit to the Fermi arc. **b,c,** Same measurements as (**a**), but for UD74K and UD78K, respectively.

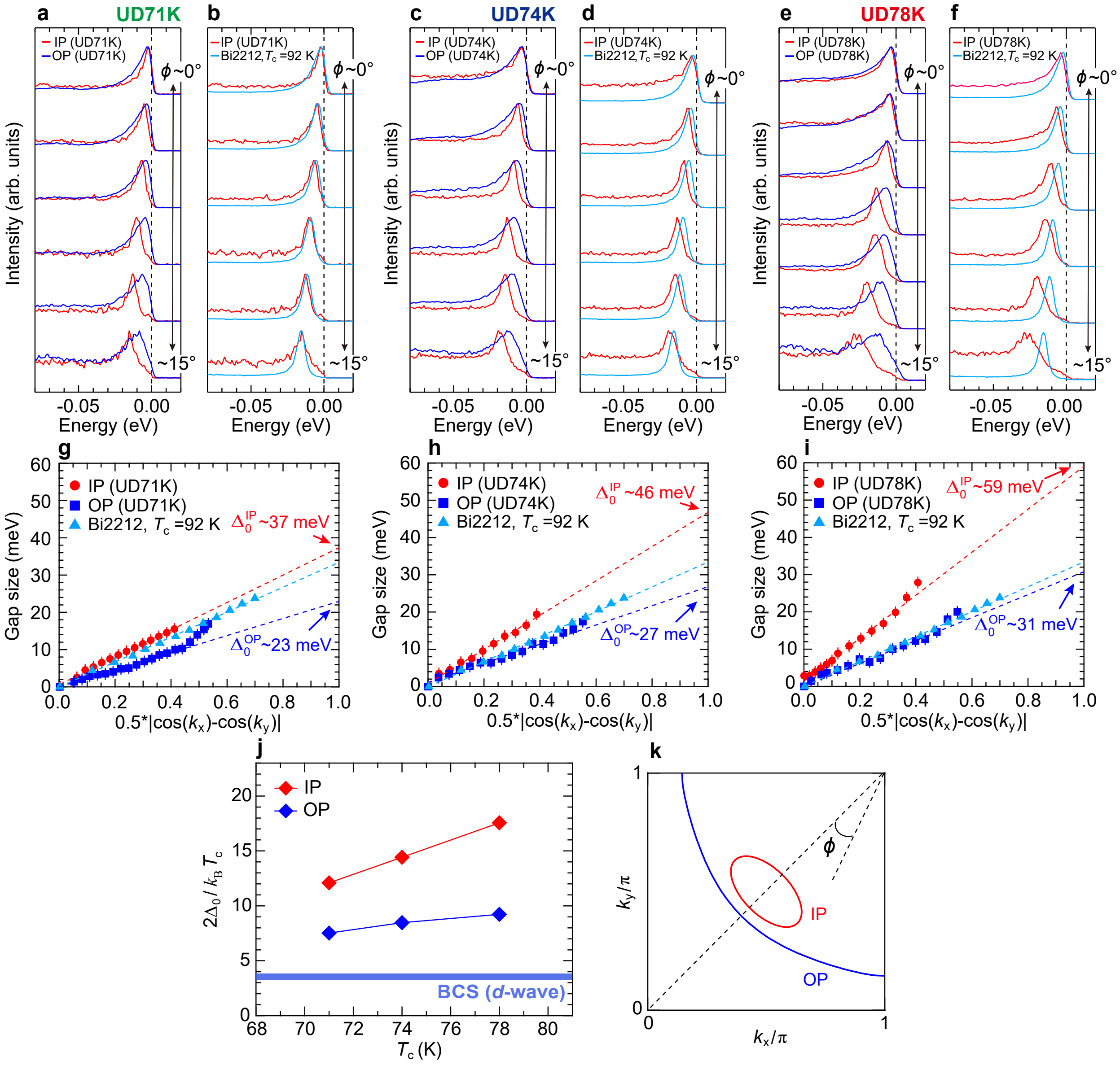

FIG. S6. **Momentum-dependence of the superconducting gap for three samples. a,** Comparison of the SC gap between the IP and OP in UD71K. EDCs taken at a similar Fermi surface angle ϕ are overlapped. **b,** Similar to **a**, but showing a comparison between the IP of UD71K and optimally doped Bi2212 [3]. **c-f,** Same measurements as in (**a,b**), but for UD74K and UD78K, respectively. **g,** Momentum dependence of the SC gap for each CuO_2 plane in UD71K and Bi2212, plotted as a function of d-wave form, $|\cos(k_x) - \cos(k_y)|/2$. The antinodal gap Δ_0 is obtained by extrapolating the gap values near the nodal region. **h,i,** Similar analysis to (**g**), but for UD74K and UD78K, respectively. **j,** Ratio $2\Delta_0/k_B T_c$ for each CuO_2 plane in our four-layer samples. The thick horizontal blue line indicates the BCS weak-coupling prediction for the d-wave superconductivity [5]. **k,** Schematic illustration of the Fermi surfaces for the IP and OP.

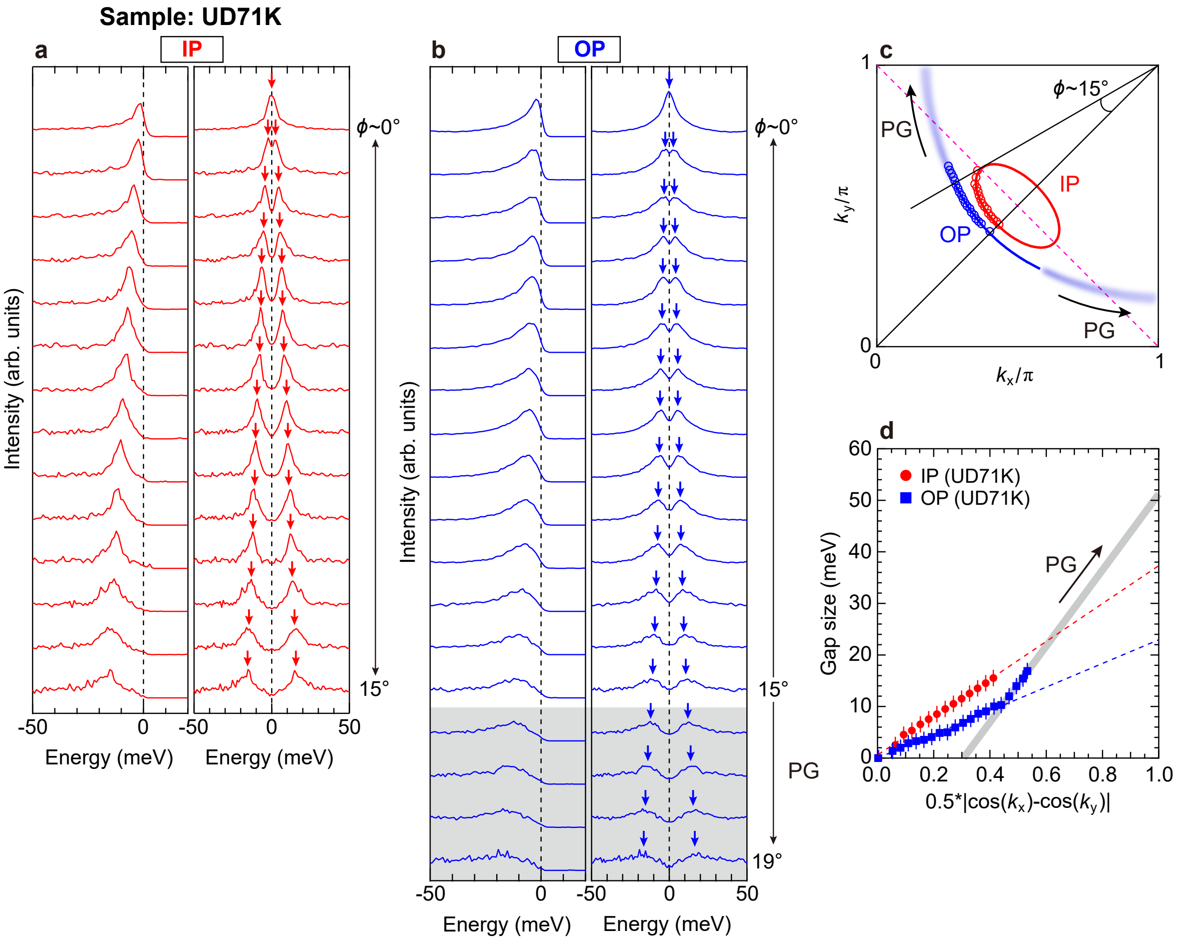

FIG. S7. **Comparison of Bogoliubov quasiparticle peaks between the pocket band in IP and the arc band in OP. a,** Raw (left panel) and symmetrized (right panel) EDCs of UD71K measured along the small Fermi pocket in the IP, from the nodal point to the pocket tip. **b,** Same as (**a**), but measured along the large Fermi surface in OP, from the nodal region toward the antinodal region. The spectra in (**a,b**) were normalized to the integrated area over the energy range from -50 meV to 50 meV. They were then plotted with the same vertical offset. The spectra in a grey shaded area correspond to the spectra that deviate from the *d*-wave form, as shown in (**d**), indicating a pseudogap (PG) feature. **c,** Fermi surface of UD71K. Red and blue circles mark the k_F points corresponding to the spectra in (**a,b**), respectively. The blue-shaded line indicates the momentum region where broad spectra due to the pseudogap are observed. **d,** Energy gap symmetry plotted with respect to the *d*-wave form. The pseudogap with a larger energy scale than the SC gap causes a deviation from the simple *d*-wave function, as indicated by a gray-shaded line.

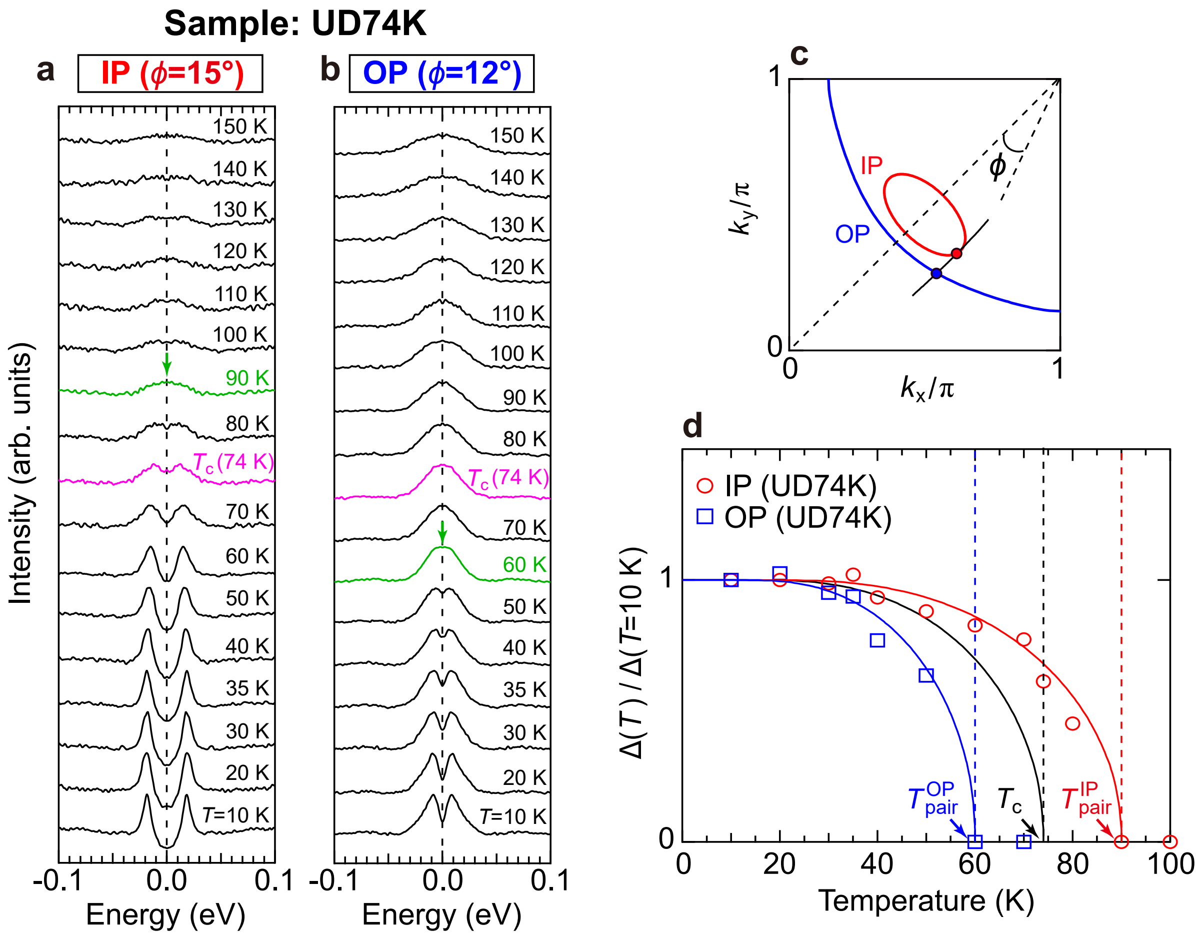

FIG. S8. **Temperature dependence of the spectral gap in the pocket band (IP) and arc band (OP), and estimation of their gap-closing temperatures. a,** Symmetrized EDCs at the tip of the small Fermi pocket in IP ($\phi = 15°$, red circle in (**c**)), measured over a wide range of temperature. The spectrum at the T_c and gap-closing temperature is indicated in pink and green, respectively. **b,** Similar to (**a**), but measured at the Fermi arc in OP ($\phi = 12°$, blue circle in **c**). **c,** Fermi surface of UD74K determined by the tight-binding fit. The black line indicates the measured momentum cut, crossing the k_F points where the EDCs in (**a,b**) were obtained. **d,** The energy gap evolution with temperature for IP and OP. The gap sizes are normalized by those at the lowest measurement temperature ($T = 10$ K). The black curve represents a BCS-type gap function with T_c =74 K. The red and blue curves represent BCS-type gap functions fitted to the data, which extrapolate the gap-closing temperatures (T_{pair}) of each CuO_2 plane marked by coloured arrows.

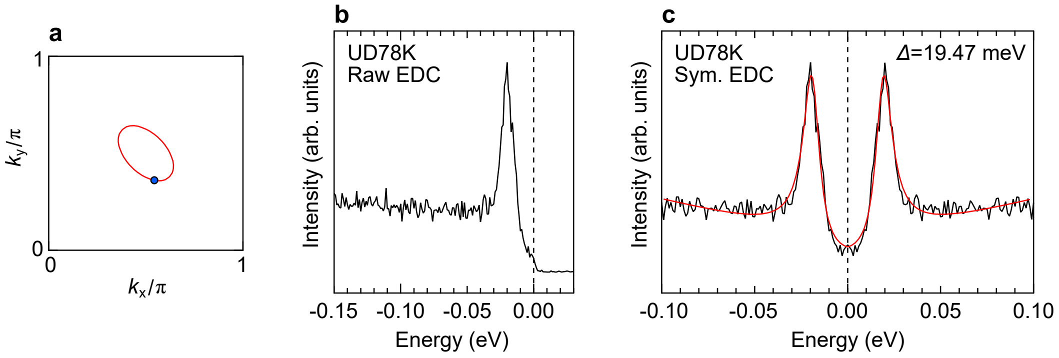

FIG. S9. **Superconducting gap fitting by phenomenological model. a,** Schematic illustration of IP in F0234. The blue circle indicates the k_F point where the data shown in (**b** and **c**) were acquired. **b, c,** Raw and symmetrized (Sym.) EDCs of UD78K in the superconducting state ($T = 10$ K), taken at the momentum point shown in (**a**). The red solid curve indicates the fitting by the phenomenological model [2].

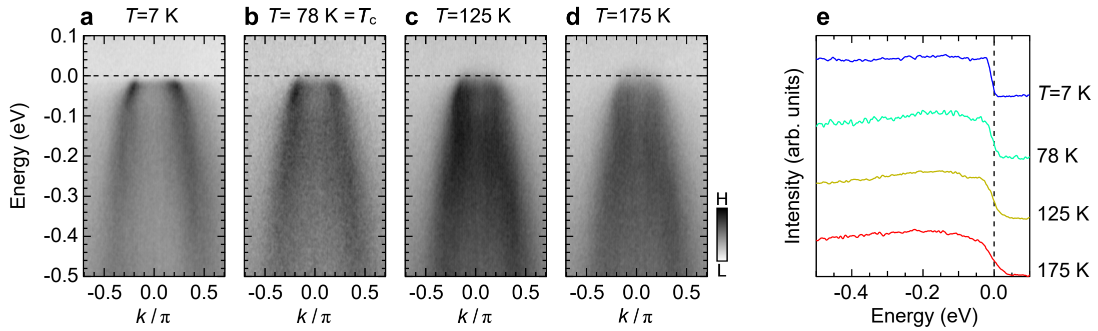

FIG. S10. **Temperature-dependence of the Bogoliubov flat band. a-d,** ARPES energy-momentum dispersions of UD78K along AFZB direction, measured at various temperatures. **e,** EDCs at the pocket center ($k = 0$) from the dispersions in (**a-d**).

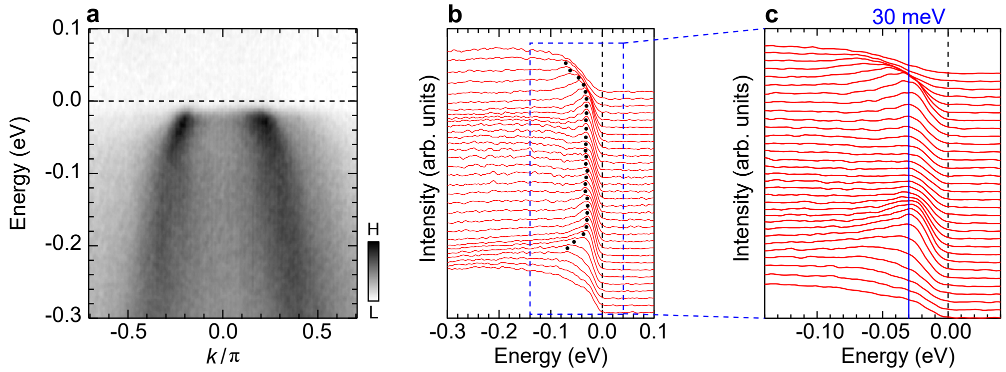

FIG. S11. **Gap size of UD78K demonstrated by using momentum cut along AFZB. a,** ARPES dispersion of UD78K, measured along AFZB. **b,** Set of EDCs, taken from $k/\pi = [-0.33, 0.36]$ in (**a**). **c,** The same data as (**b**), but zoomed in near E_F (blue dashed box in (**b**)). The gap magnitude is estimated to be ~ 30 meV (blue line).

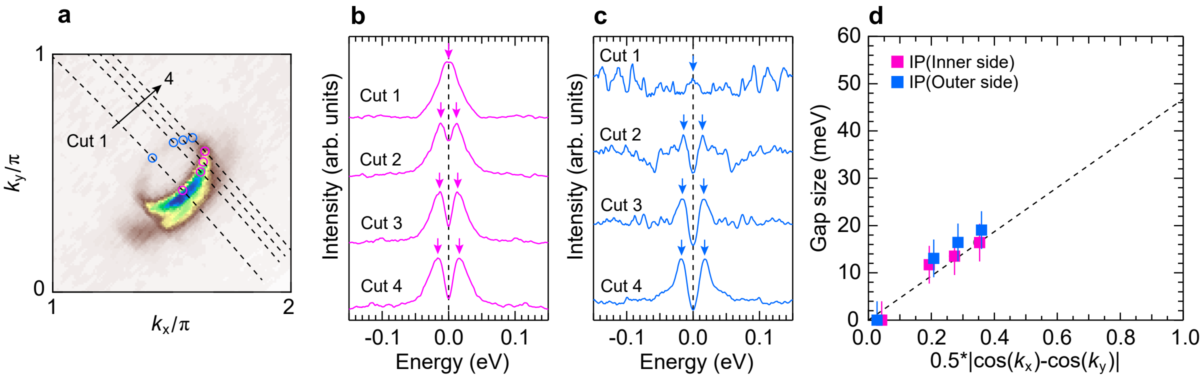

FIG. S12. ***d*-wave superconducting gap symmetry on the outer side of the small Fermi pocket.** **a,** ARPES Fermi surface of UD74K. The dashed lines and colored circles represent momentum cuts and k_F points to acquire the data shown in (**b, c**). **b,** Symmetrized EDCs of the inner side of the pocket in IP, from near-node (cut 1) to the pocket tip (cut 4), measured at the k_F points shown in (**a**) (magenta circles). **c,** Similar to (**b**), but for the outer side of the pocket (blue circles in (**a**)). **d,** Momentum-dependence of the SC gaps shown in (**b, c**), plotted as a function of *d*-wave form. The dashed line is a guideline for the gap magnitude at the antinode (Δ_0, see Fig. S6)

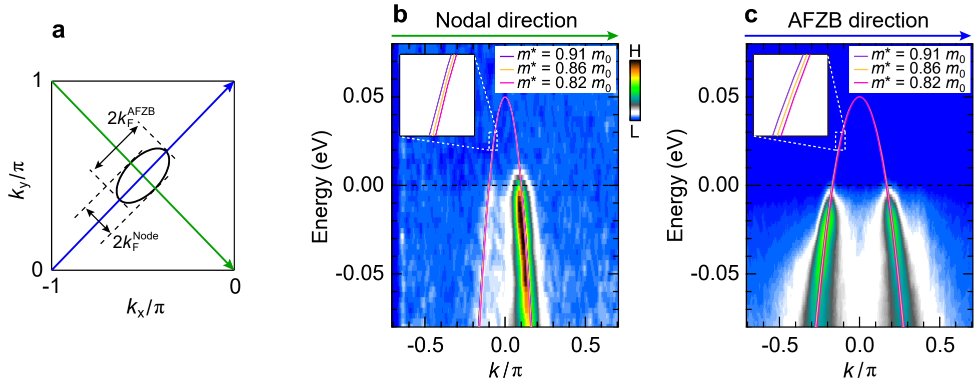

FIG. S13. **Effective masses of the small Fermi pocket: comparison between ARPES and quantum oscillation measurements.** **a,** A Schematic illustration of the elliptical Fermi pocket. The green and blue arrows show the momentum cuts used in (**b, c**), respectively. **b,** ARPES dispersion of UD74K, along the nodal direction (green arrow in (**a**)). The colored curves are the parabolic band dispersions calculated by using the effective masses obtained from the quantum oscillation measurements. The inset figure (top left) is an enlarged plot of the parabolic bands (white dashed box), demonstrating that the expected slope change is very small. **c,** Similar to (**b**), but for the AFZB direction cut (blue arrow in (**a**)).

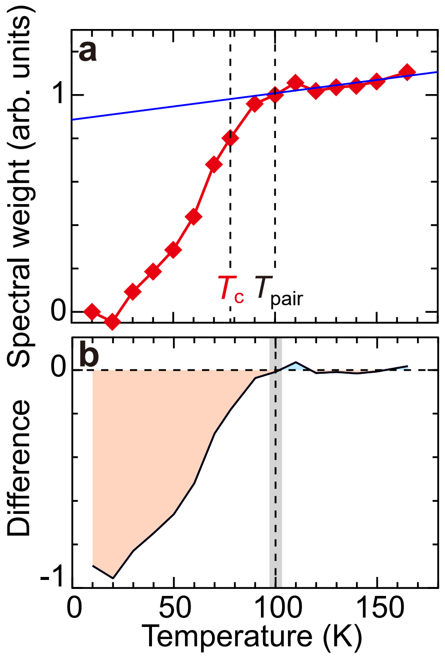

FIG. S14. **Temperature-dependent spectral-weight-loss analysis for UD78K. a,** Temperature-dependent spectral weight of UD78K, integrated near E_F (same plot shown in Fig. 3e). **b,** Difference plot of (**a**), obtained by subtracting the data points (red diamonds in (**a**)) from the linear fit (blue line in **a**). The grey region corresponds to ±3 K error range around T_{pair}.
